# Dietary and lifestyle oxidative balance score was negatively associated with the risk of diabetic kidney disease: NHANES 2005–2020

**DOI:** 10.1007/s00592-024-02399-7

**Published:** 2024-12-28

**Authors:** Yanhong Lu, Kai Huang, Youjuan Fu, Xiaoyan Huang, Ken Chen, Qiaojun Zheng, Guangda Xiang, Ling Yue

**Affiliations:** 1 Department of Endocrinology, General Hospital of Central Theater Command, Wuhan, Hubei People’s Republic of China; 2 Department of Gastroenterology, General Hospital of Central Theater Command, Wuhan, Hubei People’s Republic of China

**Keywords:** Oxidative balance score, DKD, NHANES, Dietary, Lifestyle

## Abstract

**Aims:**

There is a potential association between oxidative stress and the development of diabetic kidney disease (DKD). The Oxidative Balance Score (OBS), derived from dietary and lifestyle factors, acts as a comprehensive marker of oxidative stress. Research examining the relationship between OBS and DKD is scarce. This study aims to evaluate the association between OBS and the risk of DKD among U.S. adults.

**Methods:**

This study enrolled 6,725 eligible participants from the U.S. population through the National Health and Nutrition Examination Survey (2005–2020). Patients with DKD were defined as those with diabetes who had a urinary albumin-to-creatinine ratio ≥ 30 mg/g and/or an estimated glomerular filtration rate < 60 mL/min/1.73 m². The OBS consists of 20 composite scores derived from dietary and lifestyle factors. To assess the potential relationship between OBS and DKD, weighted logistic regression and restricted cubic spline statistical approaches were employed.

**Results:**

The risk of DKD was inversely correlated with OBS, dietary OBS, and lifestyle OBS (*p* < 0.05). Compared to the lowest quartile of OBS, the adjusted odds ratios (OR) for OBS, lifestyle OBS and dietary OBS, and DKD in the highest quartile were 0.58 (95% CI: 0.48–0.70), 0.64 (95% CI: 0.51–0.81), and 0.57 (95% CI: 0.46–0.70), respectively. A substantial nonlinear relationship between lifestyle OBS and DKD was identified using the RCS curve (p for nonlinearity = 0.0081), which appeared as an inverted ‘L’ shape. Using the two-piecewise logistic regression model, a turning point in lifestyle OBS was identified at a score of 3 (*p* < 0.001).

**Conclusions:**

Among the American population, OBS and DKD are significantly negatively correlated, suggesting that maintaining a higher OBS may reduce the risk of developing DKD.

**Supplementary Information:**

The online version contains supplementary material available at 10.1007/s00592-024-02399-7.

## Introduction

Diabetic kidney disease (DKD) is a chronic kidney disease caused by diabetes mellitus (DM), and its incidence is increasing year by year. DKD may eventually progress to end-stage kidney disease and increase the patient’s risk of cardiovascular disease and death [1, 2]. Therefore, preventing and mitigating the development of DKD has become a primary research priority. A comprehensive understanding of the pathophysiological mechanisms underlying DKD and its influencing factors is crucial for preventing its onset and progression.

Enhanced oxidative stress plays a critical role in the progression of DKD at all stages [3]. In 2001, Brownlee proposed the ‘unified mechanism theory’, which holds that the classic polyol pathway, advanced glycation end-products (AGE) pathway, protein kinase C (PKC) pathway, and hexosamine pathway are all the result of excessive ROS production in the mitochondrial respiratory chain under a high-sugar environment, i.e., the common basis of high-sugar damage – oxidative stress [4]. Recent studies have also found that ROS expression is significantly upregulated in the foot cells, glomerular endothelial cells, tubular epithelial cells of DKD mice [5]. Pro-oxidants such as smoking, alcohol consumption, and obesity can produce ROS, causing oxidative stress or reducing the defensive activities of the antioxidant system. Consuming dietary antioxidants and maintaining a healthy lifestyle are essential for preventing excessive ROS accumulation [6]. Studies have found that dietary antioxidants such as vitamin C and carotenoids are inversely associated with the incidence of type 2 diabetes across various European countries [7]. Certain minerals, such as selenium and magnesium, have demonstrated beneficial effects in the treatment of DKD [8–10]. Healthy lifestyle choices, such as smoking cessation and regular exercise, help improve oxidative stress levels in the body [11] and reduce the risk of microalbuminuria [12].

The effect of a single factor on the entire oxidation/antioxidation system is naturally limited, and many studies on single factors yield contradictory results [13]. To assess the comprehensive impact of diet and lifestyle on the overall oxidative and antioxidative balance, the Oxidative Balance Score (OBS) was devised [14]. The OBS typically integrates antioxidative and pro-oxidative components of various dietary and lifestyle factors. Previous studies have found that the pathogenesis of various diseases is closely linked to OBS, including cardiovascular diseases, depression, and respiratory diseases [15–17]. However, evidence regarding the association between OBS and DKD remains limited. This study aims to evaluate the potential correlation between OBS and the likelihood of developing DKD in U.S. adults. Furthermore, we seek to provide a foundation for advocating an antioxidant-rich diet or lifestyle as a strategy for reducing the risk of DKD.

## Methods

### Sources of data and study population

**Fig. 1:**
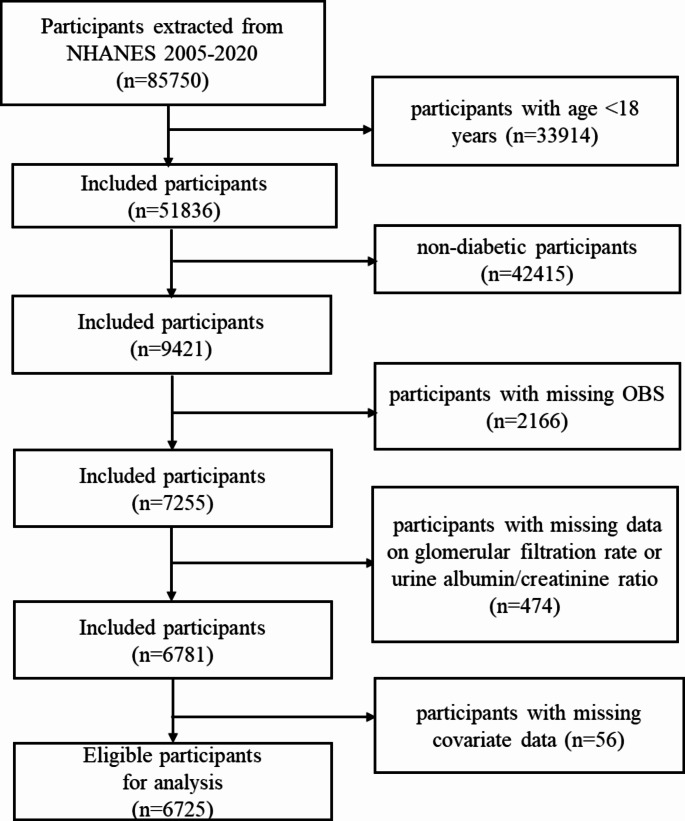
Flowchart of the sample selection from NHANES 2005–2020

For this study, demographic information was obtained from the National Health and Nutrition Examination Survey (NHANES) dataset. NHANES employs a stratified multistage sampling design, collecting data from a nationally representative sample of U.S. civilians, and is designed to monitor the health and nutritional status of adults and children across the United States. The National Center for Health Statistics (NCHS) Ethics Review Board has officially approved the NHANES survey procedure, and all participants provided written informed consent to protect their rights. Detailed statistical data can be retrieved via the following link: https://www.cdc.gov/nchs/nhanes/. All subjects in the NHANES database from 2005 to 2020, totaling 85,750 participants, were selected for this study. The following individuals were excluded: (1) aged < 18 years (*n* = 33,914); (2) nondiabetic (*n* = 42,415); (3) missing data related to OBS (*n* = 2,166); (4) missing glomerular filtration rate (eGFR) or urine albumin/creatinine ratio (UACR) (*n* = 474); (5) missing covariates (*n* = 56). The study ultimately included 6,725 eligible participants. The details of the exclusion criteria are described in Fig. 1.

### Oxidative balance score calculation

The development and calculation methodology for the OBS have been previously reported [18]. OBS is divided into dietary OBS and lifestyle OBS, representing 16 dietary nutrients and 4 lifestyle factors, respectively. OBS includes 5 pro-oxidants and 15 antioxidants, with the score positively correlated to the individual’s antioxidant activity. Dietary OBS components include dietary fiber, carotenoids, riboflavin (vitamin B2), niacin, total folate, vitamins B6, B12, C, and E, as well as calcium, magnesium, zinc, selenium, copper, iron, and total fat. Lifestyle OBS components include physical activity, body mass index (BMI), alcohol consumption, and smoking intensity. Cotinine levels measure smoking intensity, whereas alcohol consumption refers to the average daily intake over the past 12 months, including all types of alcoholic beverages. Data for physical activity were sourced from the NHANES Physical Activity Questionnaire (PAQ). Physical activity is quantified by multiplying the frequency of weekly activity occurrences by the duration of each activity and weighting it by the Metabolic Equivalent of Task (MET) score. Fat, iron, alcohol intake, cotinine levels, and BMI are categorized as pro-oxidants, while the remaining components are classified as antioxidants. Following the calculation method for OBS as outlined by Zhang et al. Alcohol consumption is classified into three categories: heavy drinkers (≥ 15 g/day for females, ≥ 30 g/day for males), non-heavy drinkers (0–15 g/day for females, 0–30 g/day for males), and non-drinkers, with corresponding scores of 0, 1, and 2. Other components are divided by sex, further grouped, and scored based on tertile weightings, with antioxidants receiving scores of 0, 1, and 2 and pro-oxidants scoring 2, 1, and 0 in the same manner. Further details are provided in Table S1. Higher OBS scores indicate greater antioxidant exposure.

### Assessment of DKD

According to the American Diabetes Association (ADA) criteria for diagnosing diabetes, individuals meeting one or more of the following conditions are considered diabetic: (1) fasting plasma glucose ≥ 7.0 mmol/L or 2-hour postprandial glucose ≥ 11.1 mmol/L; (2) random plasma glucose ≥ 11.1 mmol/L; (3) glycated hemoglobin (HbA1c) ≥ 6.5%; (4) use of diabetes medications or insulin; (5) a documented diabetes diagnosis by a healthcare professional. According to the Kidney Disease: Improving Global Outcomes (KDIGO) 2021 Guidelines, diabetes patients with an estimated glomerular filtration rate (eGFR) of less than 60 mL/min/1.73 m² or a urine albumin-to-creatinine ratio (UACR) of at least 30 mg/g are classified as having DKD.

### Covariate selection

Based on existing literature and clinical findings, covariates that might act as potential confounders in the relationship between OBS and DKD were selected: (1) Demographic variables: sex, age, ethnicity, family income-to-poverty ratio, BMI, etc.; (2) Health survey data: past illness history (hypertension, cardiovascular events, diabetes), smoking history, and OBS score; (3) Laboratory results: HbA1c, creatinine, urinary albumin, serum uric acid, UACR, eGFR. The categorization of study variables includes: sex (male and female); age (under 40, 40–59, and above 60); ethnicity (Mexican American, White, Black, and other); family income-to-poverty ratio (≤ 1 and > 1); smoking status (non-smokers, past smokers, and current smokers); alcohol consumption (heavy drinkers, moderate drinkers, and non-drinkers); cardiovascular events (presence of congestive heart failure, coronary artery disease, angina, stroke, or heart attack); hypertension (average systolic pressure ≥ 140 mmHg, diastolic pressure ≥ 90 mmHg, use of antihypertensive medication, or a diagnosis by the participant or physician); BMI (normal < 25 kg/m², overweight 25 ≤ BMI < 30 kg/m², and obese ≥ 30 kg/m²); glycemic control (HbA1c < 7% as well-controlled, HbA1c ≥ 7% as poorly controlled).

### Statistical methods

All analyses incorporated sample weights, clustering, and stratification. We utilized a one-way analysis of variance (ANOVA) for continuous variables and employed the Pearson chi-square test for categorical variables. While categorical data were reported as frequencies and percentages, continuous variables were shown as mean values accompanied by their standard deviations (SD). To verify the correlation between OBS and DKD, subjects were divided into four groups (Q1-Q4) according to the quartiles of OBS scores. The correlation between OBS and DKD was investigated using weighted logistic regression modeling. OR (odds ratio) values, 95% confidence intervals, and p-trends were calculated. Three models were developed to control for confounders. Model 1 was not adjusted for any potential confounders. Model 2 adjusted for gender, race, and age. Model 3 adjusted for smoking status, alcohol consumption, hypertension, cardiovascular events, HbA1c, and BMI based on Model 2. We analyzed the nonlinear relationship between OBS scores and DKD using restricted cubic spline curves and attempted to select the most probable threshold if it existed. The validation of the two-part logistic regression model on both sides of the inflection point was conducted. The association between OBS and DKD prevalence was investigated by two-piecewise logistic regression analysis. Finally, in the subgroup analyses, we analyzed the data according to age, gender, race, income poverty ratio, BMI, smoking status and alcohol consumption, and explored the interactions. All statistical analyses were carried out using R 4.2.1, with a statistically significant *p* < 0.05.

## Results

### Participant characteristics

In our current study, 6725 participants were recruited, including 2617 DKD patients and 4108 non-DKD patients. Table 1 describes the baseline features by quartiles: compared to participants in the lowest OBS quartile, those in the highest OBS quartile were older, had a greater proportion of White participants (65.86%), were relatively wealthier, comprised more non-smokers, and had a lower prevalence of cardiovascular disease, with comparatively lower levels of uric acid. The population was also stratified into DKD and non-DKD groups, as shown in Supplemental Table S2. Average OBS scores were lower in DKD patients compared to non-DKD participants in both dietary OBS and lifestyle OBS scores. Most DKD patients were 60 years of age or older. The number of DKD patients was higher in better-off families, and most DKD patients had comorbid hypertension (79.84%).

**Table 1:** Baseline characteristics according to the xidative balance score(OBS) quartiles

Characteristics	Q1 (4–12)	Q2 (12–18)	Q3 (18–23)	Q4 (23–35)	*P*
*Sex*					0.51
Male	938(51.44)	1015(50.18)	708(51.80)	864(53.60)	
Female	795(48.56)	901(49.82)	695(48.20)	809(46.40)	
*Age*					**0.03**
< 40	125(9.59)	133(8.49)	111(10.10)	151(9.52)	
40–59	507(37.79)	595(36.49)	469(39.92)	622(43.84)	
≥ 60	1101(52.62)	1188(55.02)	823(49.97)	900(46.65)	
*Race*					** < 0.0001**
Mexican American	226(7.02)	346(9.66)	256(9.97)	338(10.15)	
White	574(57.48)	726(64.59)	545(66.40)	637(65.86)	
Black	596(20.87)	454(12.92)	313(11.39)	312( 9.43)	
Other Race	337(14.63)	390(12.83)	289(12.24)	386(14.57)	
*Income-poverty ratio*					** < 0.0001**
≤ 1	514(22.42)	455(16.57)	254(11.54)	300(11.42)	
> 1	1219(77.58)	1461(83.43)	1149(88.46)	1373(88.58)	
*Smoke*					** < 0.0001**
Never	770(42.35)	945(50.49)	746(51.93)	945(53.58)	
Former	559(31.65)	688(35.50)	469(34.82)	544(36.08)	
Now	404(26.00)	283(14.01)	188(13.25)	184(10.34)	
*Alcohol*					** < 0.0001**
Never	898(46.11)	910(44.16)	616(36.38)	693(35.51)	
Moderate	469(28.60)	598(33.99)	464(38.44)	588(39.94)	
Heavy	366(25.29)	408(21.85)	323(25.18)	392(24.56)	
*Hypertension*					**0.002**
No	434(25.37)	567(30.22)	431(31.80)	565(34.53)	
Yes	1299(74.63)	1349(69.78)	972(68.20)	1108(65.47)	
*CVD*					** < 0.0001**
No	1182(69.96)	1410(73.74)	1096(79.58)	1369(82.11)	
Yes	551(30.04)	506(26.26)	307(20.42)	304(17.89)	
*HbA1c(%)*					0.65
< 7	1009(62.32)	1113(61.20)	801(59.23)	963(60.88)	
≥ 7	724(37.68)	803(38.80)	602(40.77)	710(39.12)	
*BMI(kg/m* ^2^ **)**					0.06
Normal weight	194(9.43)	241(9.04)	194(11.00)	230(12.80)	
Over weight	471(24.58)	543(24.87)	407(26.90)	507(26.34)	
Obesity	1068(65.99)	1132(66.09)	802(62.10)	936(60.86)	
*Uric acid(umol/L)*	345.00(291.50,416.40)	339.00(285.50,398.50)	327.10(273.60,392.60)	321.20(273.60,386.60)	** < 0.0001**
*Albumin urine(mg/L)*	16.90(7.10,46.90)	12.50(6.00,34.80)	12.00(6.00,30.40)	9.90(4.80,24.20)	** < 0.0001**
*Creatinine urine (mg/dL)*	115.00(72.00,172.00)	108.00(69.00,164.00)	101.00(63.00,151.00)	96.00(63.00,144.00)	** < 0.0001**
*uACR(mg/g)*	13.37(6.93,46.46)	11.55(6.16,31.49)	11.51(6.00,30.86)	9.60(5.67,24.04)	** < 0.0001**
*eGFR(mL/min/1.73m* ^2^ **)**	84.03(63.00,100.67)	83.84(65.73, 99.75)	88.62(69.91,102.62)	89.54(73.38,101.64)	** < 0.0001**
*OBS*	10.00( 8.00,11.00)	16.00(14.00,17.00)	21.00(20.00,22.00)	27.00(25.00,29.00)	** < 0.0001**
*Dietary OBS*	6.00( 5.00, 8.00)	12.00(11.00,14.00)	18.00(16.00,19.00)	23.00(22.00,25.00)	** < 0.0001**
*Lifestyle OBS*	3.00(2.00,4.00)	4.00(3.00,5.00)	4.00(2.00,5.00)	4.00(3.00,5.00)	** < 0.0001**

Bold values are statistically significant (*P* < 0.05)

### OBS is negatively correlated with DKD

Multiple large-scale investigations have established the undeniable impact of individual pro-oxidants or antioxidants on DKD [19, 20]. Therefore, we became interested in the association between DKD and OBS, a comprehensive measure of an individual’s oxidative stress level. The relationship between OBS and DKD is illustrated in Table 2, where weighted logistic regression analysis revealed a negative association between OBS and DKD. n Model 3, after adjusting for sex, age, ethnicity, income-to-poverty ratio, smoking status, drinking status, the presence of hypertension, cardiovascular events, HbA1c levels, and BMI, the odds ratios (ORs) and 95% confidence intervals (CIs) for DKD from the lowest to highest OBS index quartiles with OBSQ1 as the reference were: Q2 OR = 0.76 (0.62, 0.92), Q3 OR = 0.76 (0.59, 0.97), Q4 OR = 0.58 (0.48, 0.70), with *p* for trend < 0.001. This signifies that for OBS scores between 23 and 35, for every unit increase in the OBS index, the risk of DKD decreases by 42%. The OBS score was inversely correlated with the risk of DKD, indicating that an increasing OBS score is associated with a gradually declining risk of DKD, with *p* for trend < 0.0001. Similar relative stability across the models was maintained in both the partially adjusted model (Model 2) and the unadjusted model (Model 1).

**Table 2:** OR estimates for associations between OBS and DKD

	Q1	Q2	*P*	Q3	*P*	Q4	*P*	*P*-trend
*OBS*								
Model 1	1.00	0.76(0.62,0.92)	**0.01**	0.70(0.55,0.89)	**0.003**	0.51(0.42,0.62)	< 0.001	< 0.001
Model 2	1.00	0.73(0.60,0.89)	**0.002**	0.70(0.55,0.90)	**0.005**	0.53(0.44,0.64)	< 0.001	< 0.001
Model 3	1.00	0.76(0.62,0.92)	**0.01**	0.76(0.59,0.97)	**0.03**	0.58(0.48,0.70)	< 0.001	< 0.001
*OBS.dietary*								
Model 1	1.00	0.70(0.57,0.84)	**< 0.001**	0.63(0.52,0.77)	**< 0.001**	0.50(0.40,0.62)	< 0.001	< 0.001
Model 2	1.00	0.69(0.57,0.85)	**< 0.001**	0.64(0.53,0.79)	**< 0.001**	0.54(0.44,0.67)	< 0.001	< 0.001
Model 3	1.00	0.69(0.56,0.85)	**< 0.001**	0.66(0.54,0.81)	**< 0.001**	0.57(0.46,0.70)	< 0.001	< 0.001
*OBS.lifestyle*								
Model 1	1.00	1.00(0.83,1.22)	0.97	0.88(0.73,1.07)	0.19	0.70(0.58,0.85)	< 0.001	< 0.001
Model 2	1.00	0.96(0.78,1.18)	0.69	0.78(0.64,0.96)	**0.02**	0.60(0.49,0.74)	< 0.001	< 0.001
Model 3	1.00	0.97(0.79,1.19)	0.78	0.87(0.69,1.08)	0.20	0.64(0.51,0.81)	< 0.001	< 0.001

Bold values are statistically significant (*P* < 0.05)

Model 1: unadjusted model

Model 2: adjusting for sex, age, ethnicity

Model 3: adjusting for sex, age, ethnicity, income-to-poverty ratio, smoking status, drinking status, the presence of hypertension, cardiovascular events, HbA1c levels, and BMI

The specific range for the quantiles: OBS: Q1 [4, 12]; Q2 (12,18]; Q3 (18,23]; Q4 (23,35]; dietary OBS: Q1 [2, 9]; Q2 (9,14]; Q3 (14,20]; Q4 (20,31]; lifestyle OBS: Q1 [0,3]; Q2 (3,4]; Q3 (4,5]; Q4 (5,7]

Further segmentation of OBS into dietary OBS and lifestyle OBS was conducted to assess their correlations with DKD independently. For dietary OBS, a significant negative correlation with the risk of DKD was identified, remaining statistically significant after adjusting for all variables. The risk of DKD progressively decreased with higher dietary OBS scores: Q2 OR = 0.69 (0.56, 0.85), Q3 OR = 0.66 (0.54, 0.81), and Q4 OR = 0.57 (0.46, 0.70). The test for trend confirmed the statistical significance of this decreasing trend. After adjusting for all confounding factors, a higher lifestyle OBS was also strongly linked to a decreased risk of DKD (OR = 0.64 (0.51, 0.81), *p* < 0.001). Estimation of the variation in risk of DKD from OBS, dietary OBS, and lifestyle OBS in this multivariable analysis resulted in low pseudo-R2 values of 0.136, 0.136, 0.137 (Nagelkerke), respectively.

### Lifestyle OBS has a non-linear negative correlation with DKD

**Fig. 2:**
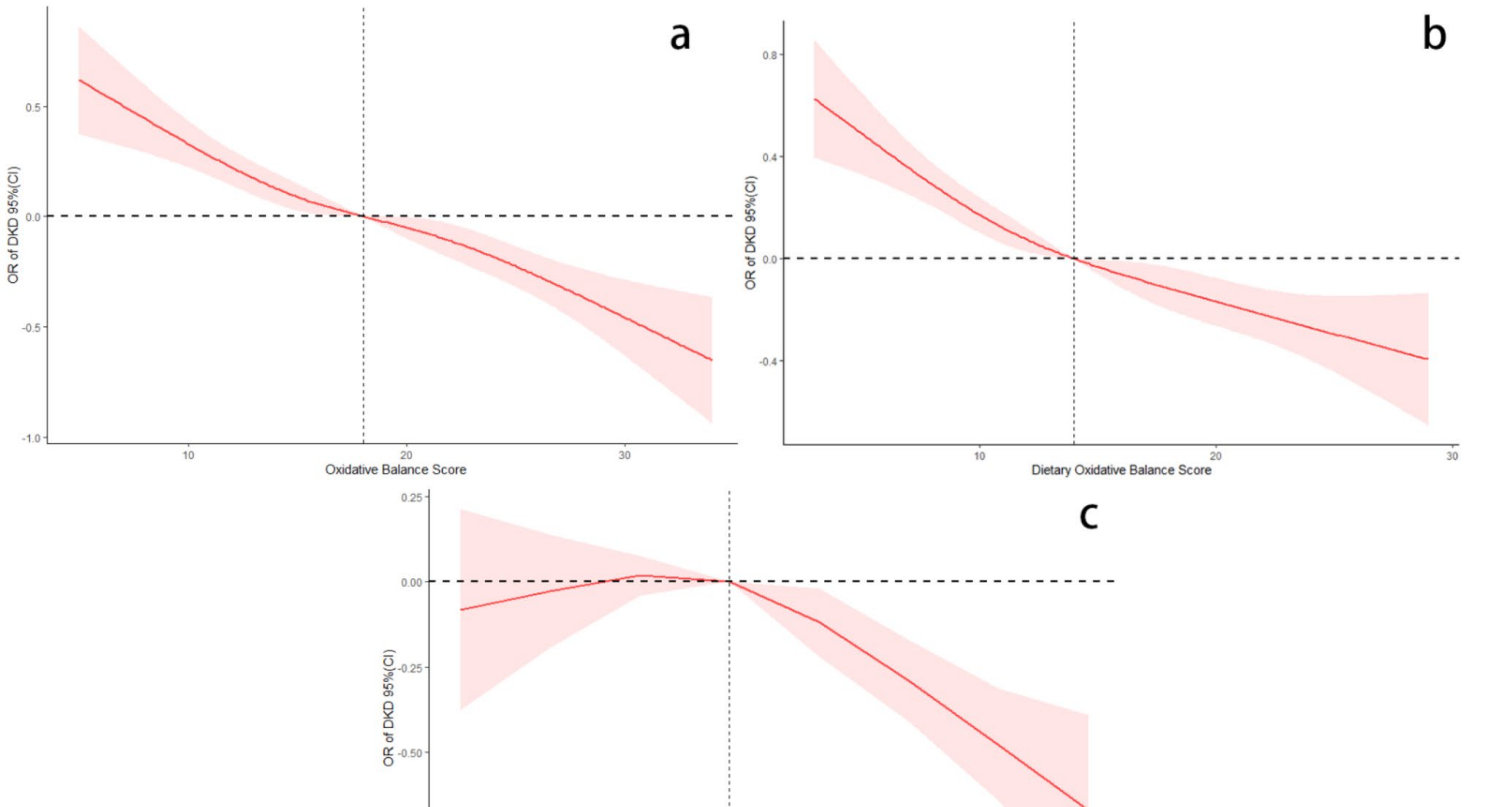
Association between OBS and DKD risk in patients with diabetes. **a** OBS and DKD risk (*p* for nonlinear = 0.4847); **b** Dietary OBS and DKD risk (*p* for nonlinear = 0.1072); **c** Lifestyle OBS and DKD risk (*p* for nonlinear = 0.0081). Adjusted for sex, age, ethnicity, income-to-poverty ratio, smoking status, drinking status, the presence of hypertension, cardiovascular events, HbA1c levels, and BMI. The solid line and red area represent the estimated values and their corresponding 95% CIs, respectively

Based on the above analysis, we identified a significant negative correlation between OBS and DKD. Therefore, we aimed further to explore the specific dose-response relationship between OBS and DKD. The multivariable-adjusted RCS analyses were conducted to evaluate this association. No substantial non-linear correlation between OBS and DKD was observed (*p* for nonlinear = 0.4847, Fig. 2a), and a linearly decreasing trend of OR with increasing total OBS index was identified. Similarly, in dietary OBS, no non-linear correlation with DKD was detected (*p* for nonlinear = 0.1072, Fig. 2b), with higher dietary OBS scores correlating with a linear decline in DKD risk. Interestingly, after adjusting for all confounders, a significant non-linear association was observed between lifestyle OBS and DKD (*p* for nonlinear = 0.0081, Fig. 2c), which appeared as an inverted ‘L’ shape. We fitted the association between lifestyle OBS and DKD using a weighted logistic regression model and a two-piecewise logistic regression model. Based on the two-piecewise logistic regression model, a turning point in lifestyle OBS was established at a score of 3 (*p* < 0.001, Table 3). After adjusting for all confounders, a slight increase in DKD risk was observed with elevated lifestyle OBS scores below 3. Conversely, once lifestyle OBS was greater than 3, a statistically significant inverse association was found between lifestyle OBS score and DKD risk, with each unit increase in lifestyle OBS score reducing the risk by approximately 19%, OR = 0.81 (0.75, 0.89).

**Table 3:** Threshold effect analysis of OBS and DKD risk

	Adjusted OR(95% CI)
*Lifestyle OBS*	
Fitting model by multivariate logistic regression	
*Total*	0.87 (0.83,0.92)
Fitting model by two-piecewise logistic regression	
*Inflection point*	3.00
< Inflection point	1.06 (0.80,1.40)
≥Inflection point	0.81 (0.75,0.89)

The overall trend in the relationship between the Obesity Index (OBS) and Diabetic Kidney Disease (DKD) risk persisted despite slight variations in results from linear and nonlinear analyses (restricted cubic spline curves). Suggests that higher OBS, particularly lifestyle OBS scores above the inflection point, are significantly associated with lower odds of developing DKD.

### Subgroup analysis of factors correlated with the association between OBS and DKD

To ensure the credibility of our research conclusions, we performed a subgroup analysis to explore other relevant factors that may influence the connection between the OBS and DKD. Table 4 presents the multiple logistic regression relationships between OBS and DKD across various subgroups. A significantly lower risk of DKD was correlated with higher OBS in female diabetes patients (OR = 0.66, 95% CI: 0.50–0.88), with an even more pronounced effect observed in males (OR = 0.53, 95% CI: 0.40–0.69). Among diabetes patients aged ≥ 60 years, increasing OBS was linked to a decreasing trend in the risk of DKD. Maintaining an OBS in the highest quartile could reduce the risk of DKD by 55% (OR = 0.45, 95% CI: 0.35–0.59).

**Table 4:** The association between OBS and DKD in subgroup analysis

	Q1	Q2	*P*	Q3	*P*	Q4	*P*	*P* interaction
*Sex*								0.71
Male	1.00	0.67(0.51,0.89)	**0.01**	0.70(0.50,0.98)	**0.04**	0.53(0.40,0.69)	**< 0.001**	
Female	1.00	0.86(0.65,1.14)	0.28	0.85(0.63,1.14)	0.27	0.66(0.50,0.88)	**0.01**	
*Age*								0.05
<40	1.00	1.10(0.52,2.33)	0.79	0.99(0.50,1.96)	0.99	1.21(0.61,2.41)	0.57	
40–59	1.00	1.05(0.72,1.54)	0.79	1.04(0.64,1.68)	0.88	0.71(0.48,1.05)	0.08	
≥ 60	1.00	0.58(0.45,0.74)	**< 0.001**	0.60(0.46,0.79)	**< 0.001**	0.45(0.35,0.59)	**< 0.001**	
*Race*								0.08
Mexican American	1.00	1.00(0.57,1.76)	1.00	0.93(0.50,1.74)	0.82	0.74(0.45,1.22)	0.23	
White	1.00	0.65(0.48,0.87)	**0.005**	0.63(0.44,0.90)	**0.01**	0.44(0.34,0.59)	**< 0.001**	
Black	1.00	0.80(0.58,1.09)	0.16	0.96(0.66,1.38)	0.81	0.85(0.61,1.17)	0.32	
Other race	1.00	0.98(0.62,1.53)	0.92	1.08(0.65,1.80)	0.75	1.02(0.61,1.71)	0.94	
*Income-poverty ratio*								0.07
≤ 1	1.00	0.94(0.63,1.40)	0.76	0.81(0.54,1.22)	0.31	0.98(0.64,1.49)	0.91	
>1	1.00	0.71(0.57,0.90)	**0.01**	0.73(0.56,0.96)	**0.03**	0.52(0.41,0.67)	**< 0.001**	
*Smoke*								0.24
Never	1.00	0.81(0.61,1.09)	0.16	0.83(0.61,1.12)	0.23	0.62(0.48,0.81)	**< 0.001**	
Former	1.00	0.60(0.42,0.84)	**0.004**	0.59(0.41,0.85)	**0.005**	0.41(0.29,0.59)	**< 0.001**	
Current	1.00	0.92(0.61,1.38)	0.68	0.80(0.46,1.37)	0.41	1.06(0.64,1.76)	0.81	
*Alcohol*								0.23
Never	1.00	0.69(0.52,0.92)	**0.01**	0.71(0.54,0.95)	**0.02**	0.53(0.41,0.69)	**< 0.001**	
Moderate	1.00	0.78(0.54,1.11)	0.16	1.00(0.63,1.61)	0.99	0.66(0.46,0.95)	**0.02**	
Heavy	1.00	0.94(0.62,1.41)	0.76	0.58(0.36,0.93)	**0.02**	0.62(0.40,0.95)	**0.03**	
*Hypertension*								0.07
No	1.00	1.14(0.77,1.67)	0.51	0.98(0.62,1.56)	0.95	0.66(0.46,0.95)	**0.03**	
Yes	1.00	0.65(0.53,0.81)	**< 0.001**	0.68(0.52,0.90)	**0.01**	0.55(0.44,0.69)	**< 0.001**	
*CVD*								0.41
No	1.00	0.85(0.66,1.09)	0.20	0.81(0.61,1.09)	0.17	0.65(0.50,0.84)	**0.001**	
Yes	1.00	0.58(0.40, 0.86)	**0.01**	0.64(0.42, 0.98)	**0.04**	0.46(0.29, 0.72)	**< 0.001**	
*HbA1c(%)*								0.06
<7	1.00	0.78(0.60,1.01)	0.06	0.72(0.53,0.97)	**0.03**	0.49(0.38,0.61)	**< 0.001**	
≥ 7	1.00	0.70(0.53,0.91)	**0.01**	0.81(0.57,1.16)	0.26	0.70(0.51,0.95)	**0.02**	
*BMI(kg/m* ^**2**^ **)**								0.33
Normal weight	1.00	0.61(0.38, 0.99)	**0.04**	0.60(0.32, 1.12)	0.11	0.50(0.29, 0.85)	**0.01**	
Over weight	1.00	0.86(0.58,1.26)	0.43	0.63(0.41,0.96)	**0.03**	0.47(0.31,0.70)	**< 0.001**	
Obesity	1.00	0.74(0.57,0.96)	**0.03**	0.84(0.61,1.15)	0.27	0.63(0.49,0.80)	**< 0.001**	

Bold values are statistically significant (*P* < 0.05)

For families with favorable economic conditions, a higher OBS was closely associated with a lower chance of developing DKD (OR = 0.52, 95% CI: 0.41–0.67), with similar trends observed in patients with hypertension or cardiovascular events. Irrespective of glycated HbA1c and BMI values, maintaining an OBS in the highest quartile is associated with a lower risk of DKD.

Furthermore, interaction tests indicated that the association between the OBS and DKD did not exhibit significant differences across stratified layers. This suggests no significant interaction in the negative correlation between OBS and factors such as age, ethnicity, gender, BMI, poverty ratio, smoking status, drinking status, hypertension, cardiovascular events, or HbA1c levels (all interaction tests *p* > 0.05). This consistency across different strata implies that the negative correlation between OBS and DKD risk remains stable across various demographic and clinical characteristics.

## Discussion

Through a retrospective cross-sectional analysis of 6,725 individuals from the NHANES database, we found that total OBS, dietary OBS, and lifestyle OBS are all negatively associated with DKD. We employed restricted cubic spline (RCS) regression to examine the non-linear relationship between OBS and DKD. Interestingly, we found a significant inverse L-shaped non-linear association between lifestyle OBS and DKD. We identified a turning point at a lifestyle OBS of 3 using the threshold effect, suggesting that maintaining lifestyle OBS within a specific range is more effective in combating oxidative stress, thereby reducing the risk of DKD. Our study highlights the pivotal role of antioxidant-rich diets and healthy lifestyles in preventing DKD.

Several animal experiments and clinical studies have investigated the link between dietary antioxidants and DKD. The inclusion of these dietary constituents has been incorporated into our OBS module. Studies have shown that curcumin can improve oxidative stress status in diabetic rats through the NRF2/KEAP1/ARE pathway and alleviate renal fibrosis [21]. A certain amount of selenium intake may be a crucial factor in preventing DKD. The mechanism of action may involve regulating the expression of genes that synthesize enzymes responsible for carbohydrate metabolism, thereby improving blood glucose levels and lipid profiles [22]. Curcumin can prevent kidney damage in diabetic rats by activating Nrf2 and inhibiting the PKC-β/p66Shc pathway. Antioxidant intake can reduce the expression of inflammatory factors (TGF-β1, TNF-α, IL-6, IL-10) and decrease the apoptosis of rat podocytes [23]. Recent studies suggest that higher dietary flavonoid intake is associated with a decreased risk of DKD. This association may be attributed to the antioxidant effects of flavonoids, which include reducing ROS production, inhibiting lipid peroxidation, and increasing the activity of antioxidant enzymes. Antioxidant diets can inhibit ROS-induced DNA damage, reduce cytoplasmic cytochrome c levels, increase mitochondrial membrane potential, and improve mitochondrial dysfunction [24, 25]. This finding aligns with our results indicating that dietary OBS has a negative linear association with DKD. The exact biological mechanisms underlying the link between antioxidants—encompassing both dietary and lifestyle factors—and DKD remain unclear. Antioxidants may enhance insulin receptor sensitivity, thereby improving hyperglycemic conditions in DKD patients [26]. Additionally, antioxidants can increase the activity of endogenous antioxidant enzymes, such as glutathione peroxidase and superoxide dismutase, thereby enhancing renal cellular antioxidant and detoxifying capacities. Furthermore, antioxidant intake may indirectly improve the prognosis of DKD patients by reducing inflammatory responses and enhancing endothelial cell function [27].

The progression of DKD can be influenced by adjusting the oxidative balance through lifestyle modifications. However, limited evidence currently exists regarding the non-linear relationship between lifestyle OBS and DKD. The RCS regression indicates no significant non-linear correlation between overall OBS and dietary OBS with DKD. Notably, there is a significant non-linear correlation between lifestyle OBS and DKD. Our findings suggest that individuals with a lifestyle OBS greater than 3 have a lower risk of DKD. For every unit increase in OBS, the risk of developing DKD decreases by approximately 19% (OR = 0.81, 95% CI = 0.75–0.89). Oxidative stress may predominate at low lifestyle OBS levels, suggesting that lifestyle changes significantly impact DKD only once a certain threshold is achieved. Smoking is a significant factor in the lifestyle OBS. Research by Jiang et al. found that smoking increases the risk of DKD in diabetic patients by 49% [28]. Studies have found that quitting smoking in patients with DKD significantly decreases proteinuria and delays DKD progression [29]. Our study also demonstrates that diabetic patients who quit smoking and maintain high OBS scores can reduce the DKD risk by 59% (OR = 0.41, 95% CI = 0.29–0.59). Obesity is a significant risk factor for DKD. A high lifestyle OBS score reflects increased physical activity and a more favorable BMI. A systematic review by Zoccali concluded that weight loss significantly reduces the urine albumin-to-creatinine ratio and restores the glomerular filtration rate in CKD patients [30]. Physical activity can improve insulin sensitivity [31], reduce nitric oxide (NO) degradation by increasing NO generation, and decrease ROS production, thus enhancing glomerular filtration function and ameliorating glomerulosclerosis and tubulointerstitial fibrosis [32]. Regular moderate-intensity physical activity can reduce the incidence and slow the progression of renal disease in patients at risk for DKD or those already diagnosed [33, 34]. The lifestyle OBS encompasses various factors, including physical activity, diet, and smoking status. Our study provides new evidence that maintaining a higher lifestyle OBS is beneficial in reducing the risk of developing DKD.

Diet and lifestyle intervention are a consistent approach to DKD management. The 2022 Diabetes Management in Chronic Kidney Disease: A Consensus Report by the ADA and KDIGO reiterates the importance of dietary intervention, recommending a personalized and balanced diet rich in vegetables, fruits, and whole grains. In terms of exercise, the consensus recommends moderate to high-intensity or vigorous physical activity while avoiding sedentary behaviors. The report also strongly advocates for smoking cessation. However, existing guidelines for comprehensive lifestyle intervention management in DKD patients lack focus on the impact of an antioxidant-rich diet and lifestyle, and do not assess whether these dietary and lifestyle interventions meet established standards. Our research indicates that a diet rich in antioxidants and effective lifestyle management benefit the management of DKD, guiding future dietary and lifestyle interventions for patients. The OBS can assist in assessing whether the antioxidant-rich diet and lifestyle interventions of DKD patients meet established standards, enabling physicians to provide more accurate personalized medical guidance.

Our research demonstrated that increased intake of antioxidants (including dietary and lifestyle) may reduce the risk of developing DKD in adults. Nevertheless, it is important to acknowledge the limitations of this study. The retrospective cross-sectional design limits the ability to establish causality between the OBS and DKD. Additionally, although adjustments were made for potential covariates, we cannot entirely exclude the influence of residual confounding factors. Furthermore, because the cohort consisted solely of American citizens, further research is necessary to determine whether these findings are applicable to diverse populations. In the future, we aim to apply the OBS score to a larger cohort of DKD patients and further investigate its implications.

## Conclusion

In summary, an analysis of nationally representative samples of American adults indicates that the OBS has a substantial inverse association with the risk of DKD. Additionally, the relationship between lifestyle factors incorporated into the OBS and DKD is nonlinear, with a higher OBS indicating a reduced risk of DKD. These findings suggest that antioxidant-rich diets and lifestyles may provide significant benefits for the prevention and management of DKD. Future research should focus on investigating the specific causal links and potential mechanisms between the OBS and DKD.

## Supplementary Information

Below is the link to the electronic supplementary material.

Supplementary file1 (DOCX 19 kb)

Supplementary file2 (DOCX 21 kb)

Supplementary file3 (DOCX 12 kb)

## Data Availability

Data described in the manuscript, code book, and analytic code will be made available upon request pending application and approval.
