# Supplementary material for: Dietary and lifestyle oxidative balance score was negatively associated with the risk of diabetic kidney disease: NHANES 2005–2020
Source: Acta Diabetol. 2024 Dec 28;62(6):819–29. doi: 10.1007/s00592-024-02399-7 (PMC12141365; doi:10.1007/s00592-024-02399-7)
Supplement: Supplementary file 2 — Supplementary file2 (DOCX 21 kb) [file 592_2024_2399_MOESM2_ESM.docx]

**Table S2 baseline characteristics**

| **Characteristics** | **Non-DKD**  **(n=4108)** | **DKD**  **(n=2617)** | ***P*** |
| --- | --- | --- | --- |
| **Sex** |  |  | 0.92 |
| Male | 2099(51.71) | 1426(51.92) |  |
| Female | 2009(48.29) | 1191(48.08) |  |
| **Age** |  |  | **<0.001** |
| ＜40 | 393(11.04) | 127( 6.30) |  |
| 40-59 | 1619(46.28) | 574(27.34) |  |
| ≥60 | 2096(42.67) | 1916(66.36) |  |
| **Race** |  |  | 0.34 |
| Mexican American | 740(9.32) | 426(9.24) |  |
| White | 1419(63.46) | 1063(64.41) |  |
| Black | 1021(13.11) | 654(13.73) |  |
| Other Race | 928(14.11) | 474(12.62) |  |
| **Income-poverty ratio** |  |  | **0.01** |
| ≤1 | 907(14.40) | 616(16.90) |  |
| ＞1 | 3201(85.60) | 2001(83.10) |  |
| **Smoke** |  |  | **<0.001** |
| Never | 2176(52.03) | 1230(45.99) |  |
| Former | 1267(32.12) | 993(39.38) |  |
| Now | 665(15.85) | 394(14.63) |  |
| **Alcohol** |  |  | **<0.001** |
| Never | 1762(37.07) | 1355(46.59) |  |
| Moderate | 1349(36.32) | 770(33.93) |  |
| Heavy | 997(26.61) | 492(19.48) |  |
| **Hypertension** |  |  | **<0.001** |
| No | 1472(36.48) | 525(20.16) |  |
| Yes | 2636(63.52) | 2092(79.84) |  |
| **CVD** |  |  | **<0.001** |
| No | 3359(82.02) | 1698(66.54) |  |
| Yes | 749(17.98) | 919(33.46) |  |
| **HbA1c(%)** |  |  | **<0.001** |
| ＜7 | 2522(64.41) | 1364(54.50) |  |
| ≥7 | 1586(35.59) | 1253(45.50) |  |
| **BMI(kg/m^2^)** |  |  | 0.09 |
| Normal weight | 497(10.09) | 362(11.60) |  |
| Over weight | 1220(26.60) | 708(23.95) |  |
| Obesity | 2391(63.31) | 1547(64.45) |  |
| **Uric acid(umol/L)** | 321.20(273.60,380.70) | 356.90(297.40,422.30) | **<0.001** |
| **Albumin urine(mg/L)** | 8.30( 4.50, 15.20) | 55.00(18.90,160.00) | **<0.001** |
| **Creatinine urine (mg/dL)** | 106.00(68.00,161.00) | 102.00(63.00,151.00) | **<0.001** |
| **uACR(mg/g)** | 7.88( 5.25, 12.99) | 52.24(25.45,146.21) | **0.04** |
| **eGFR(mL/min/1.73m^2^)** | 91.85(78.77,103.65) | 63.77(50.59, 93.92) | **<0.001** |
| **OBS** | 19.00(14.00,25.00) | 17.00(12.00,23.00) | **<0.001** |
| **Dietary OBS** | 16.00(11.00,21.00) | 14.00( 9.00,19.00) | **<0.001** |
| **Lifestyle OBS** | 4.00(3.00,5.00) | 3.00(2.00,4.00) | **<0.001** |
