# Supplementary material for: Dietary and lifestyle oxidative balance score was negatively associated with the risk of diabetic kidney disease: NHANES 2005–2020
Source: Acta Diabetol. 2024 Dec 28;62(6):819–29. doi: 10.1007/s00592-024-02399-7 (PMC12141365; doi:10.1007/s00592-024-02399-7)
Supplement: Supplementary file 3 — Supplementary file3 (DOCX 12 kb) [file 592_2024_2399_MOESM3_ESM.docx]

**Funding:** This study was funded by International Diabetes Exchange and Practice Special Fund of China International Exchange Foundation of Chinese Medical Association (grant number Z‑2017‑26‑1902).

**Conflict of Interest:** The authors declare that they have no conflict of interest.
